# Cooling Cycle Optimization for a Vuilleumier Refrigerator

**DOI:** 10.3390/e23121562

**Published:** 2021-11-24

**Authors:** Raphael Paul, Abdellah Khodja, Andreas Fischer, Karl Heinz Hoffmann

**Affiliations:** Institut für Physik, Technische Universität Chemnitz, 09107 Chemnitz, Germany; raphael.paul@physik.tu-chemnitz.de (R.P.); akhodja@uni-osnabrueck.de (A.K.); andreas.fischer@physik.tu-chemnitz.de (A.F.)

**Keywords:** piston motion optimization, endoreversible thermodynamics, Stirling engine, irreversibility, power, efficiency, optimization

## Abstract

Vuilleumier refrigerators are a special type of heat-driven cooling machines. Essentially, they operate by using heat from a hot bath to pump heat from a cold bath to an environment at intermediate temperatures. In addition, some external energy in the form of electricity can be used as an auxiliary driving mechanism. Such refrigerators are, for example, advantageous in situations where waste heat is available and cooling power is needed. Here, the question of how the performance of Vuilleumier refrigerators can be improved is addressed with a particular focus on the piston motion and thus the thermodynamic cycle of the refrigerator. In order to obtain a quantitative estimate of the possible cooling power gain, a special class of piston movements (the AS motion class explained below) is used, which was already used successfully in the context of Stirling engines. We find improvements of the cooling power of more than 15%.

## 1. Introduction

**Figure 1:**
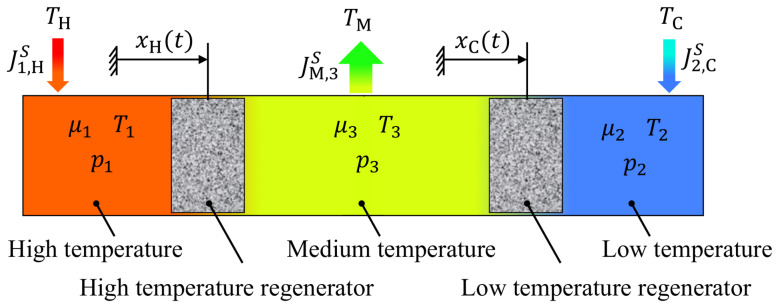
Schematics of a Vuilleumier refrigerator operating between three external heat baths with the temperatures $T_{H}>T_{M}>T_{C}.$ Cyclic changes in the size of the three subsystems 1, 2, and 3 are realized by moving the two regenerators. The state of the working gas in the three subsystems is given by the respective chemical potential μ, the temperature *T* and the pressure *p*. The regenerator movement induces pressure oscillations as well as the entropy fluxes $J_{1,H}^{S}$, $J_{M,3}^{S}$, and $J_{2,C}^{S}$.

In Vuilleumier machines [1], an enclosed working gas cyclically exchanges heat with three external heat baths at different temperatures. As schematically shown in Figure 1, the working gas is contained in three subsystems, each of which is in thermal contact with one of the baths.

The three subsystems are connected by two regenerators that act as heat capacitors. Cyclical changes in the subsystem’s volumes lead to mutual exchanges of working gas through the regenerators, which induces variations in the system’s mean temperature and pressure. In the setup considered here, the overall gas volume is kept constant, and the regenerators act as displacer pistons. The displacer movements are controlled in such a way that heat is taken from the hot external reservoir and dumped into the medium external reservoir. The respective exergy is used to drive another heat flow from the cold external reservoir to the medium external reservoir.

Vuilleumier machines are used in a variety of applications, either as heat pumps, refrigerators, or cryocoolers. As heat pumps, they can, for example, be applied in residential heating [2,3,4,5]. On the other hand, as refrigerators, they can, for example, be used for cargo cooling [2,5,6] and low-temperature cooling [7,8,9,10,11,12]. In this paper, we will focus on a Vuilleumier refrigerator.

Different approaches can be taken to optimize Vuilleumier refrigerators for maximum cooling power or maximum coefficient of performance (COP). One approach is to modify the general design concept and to optimize the associated design parameters. Another more process-oriented approach is to optimize the movements of the displacer pistons, that is, the functions $x_{H}\left(t\right)$ and $x_{C}\left(t\right)$ from Figure 1.

Corresponding approaches looking at piston path optimizations have already been used for a variety of heat-driven engines starting with investigations on Otto [13,14] and Diesel engines [15,16,17]. However, other thermodynamic cycles and engines have also been analyzed: among others, the Brayton [18,19] and the Miller [20] cycles. A thermodynamic cycle that is closely related to the Vuilleumier cycle is the Stirling cycle. Investigations into the improvement of Stirling engines by optimally controlling the piston motion were performed for different Stirling configurations, such as free-piston-type engines [21], beta-type engines [22,23], and alpha-type engines [24,25,26,27]. Here, as in [24,25], the optimizations were performed based on parametric functions (called the AS motion class), whereas in [22,26,27], indirect optimal control algorithms that are based on Pontryagin’s maximum principle were applied to find the power- or efficiency-optimal piston motion. These investigations show that significant performance improvements of Stirling engines are possible via optimizing the piston motion.

In fact, for Vuilleumier heat pumps, a piston motion [28] that strongly deviates from the typical sinusoidal motion has already been applied [29,30]. In this dwell-based piston motion (D-motion) the displacer pistons of a free-piston Vuilleumier heat pump are held in their extreme positions for defined shares of the cycle time. Chen [29] showed, with the help of a thermodynamic model, that for the parameters considered, this leads to 28% higher heat output and only slightly reduced COP.

In this investigation, we want to explore the potential cooling-power gains of a Vuilleumier refrigerator by choosing a suitable piston motion while taking into account frictional losses, finite heat transfer, and finite mass transfer. In order to obtain a model with very low numerical effort, we will use ideal regenerators. This will also help to not obscure the effects of the aforementioned loss phenomena by considering, in addition, the irreversibilities in a realistic regeneration process.

## 2. Thermodynamic Modeling of a Vuilleumier Refrigerator

### 2.1. Endoreversible Modeling

In order to determine the possible performance gains for a Vuilleumier refrigerator by using an optimized piston motion, the irreversible thermodynamics of the refrigerator needs to be captured. This especially concerns loss terms originating from finite heat and finite mass transfer as well as friction. Endoreversible Thermodynamics is a systematic approach to modeling such thermodynamic non-equilibrium systems [31,32,33]. The notion “endoreversible” that goes back to early work of Rubin [34,35] was later used by De Vos and others [36,37,38,39]. The underlying concepts of Endoreversible Thermodynamics were used successfully on a variety of thermodynamic systems, such as solar-powered heat engines [40,41,42,43], light-driven engines [44,45,46], and chemical devices [14,47,48,49,50]. A central focus has naturally been on heat engines in general [51,52,53,54], including engines under the influence of randomly varying conditions [55,56,57].

The basic idea of Endoreversible Thermodynamics is to think of a complex thermodynamic system—for instance, a power station, a full-featured chemical plant, or just a Vuilleumier refrigerator as discussed here—as a network of interacting subsystems. It is then assumed that each of these subsystems can be described as a thermodynamic equilibrium system. In the following, we will focus on two kinds of such subsystems: reservoirs and engines. These subsystems exchange thermodynamic extensities, such as volume, electric charge, entropy or streams of matter. It is only these exchanges in the form of fluxes between the subsystems that are allowed to occur in an irreversible manner. The advantage of such a decomposition of the overall system lies in the ease of the resulting system description: for the reservoirs and engines the usual thermodynamic equilibrium modeling can be used, and for the interactions well-known transport equations can be used. Thus, the entropy production—and with that, the thermodynamic performance—can be captured quantitatively.

Usually, reservoirs are depicted as rectangles in a schematic drawing of an endoreversible system. A typical example is a heat bath: if it is of infinite heat capacity, then it is characterized by its temperature; on the other hand, if it is of finite heat capacity, then we need its energy U(S) indicating how the energy content increases when entropy is added. More generally, finite reservoirs are defined by their energy function $U_{i}=U_{i}\left(X_{i}^{\alpha }\right)$, where one or several $X_{i}^{\alpha }$ are the amount of extensity α inside reservoir *i*. The respective intensity $Y_{i}^{\alpha }$ can then be obtained by using the thermodynamic potential feature of the internal energy as follows:

(1) $$\begin{array}{c} Y_{i}^{\alpha }=\frac{\partial U_{i}\left(X_{i}^{\alpha }\right)}{\partial X_{i}^{\alpha }}. \end{array}$$

The amount of extensity α present in a subsystem *i* changes when an influx $J_{i}^{\alpha }$ of that extensity occurs based on

(2) $$\begin{array}{c} {\dot{X}}_{i}^{\alpha }=\sum_{k}J_{i,k}^{\alpha }, \end{array}$$

where *k* counts the *contact points* at which the extensity enters the subsystem.

Reservoirs are usually considered to be homogeneous and thus, each intensity $Y_{i}^{\alpha }$ is the same at all contact points of a reservoir. Then, the change in energy for a reservoir follows from the Gibbs equation:

(3) $$\begin{array}{c} {\dot{U}}_{i}=\sum_{\alpha }Y_{i}^{\alpha }\;{\dot{X}}_{i}^{\alpha }=\sum_{\alpha ,k}Y_{i}^{\alpha }J_{i,k}^{\alpha }. \end{array}$$

Often, this energy flux is figuratively spoken of as being “carried” by the extensity flux.

Engines are used to describe energy conversion processes. Here, we consider only engines which have no storage capacity for extensities and thus, at each moment, for each extensity, all fluxes have to cancel and thus

(4) $$\begin{array}{c} 0=\sum_{k}J_{i,k}^{\alpha }\;\mathrm{for}\;\mathrm{all}\;\alpha . \end{array}$$

The same must hold true for the accompanying energy fluxes:

(5) $$\begin{array}{c} 0=\sum_{\alpha ,k}Y_{i,k}^{\alpha }J_{i,k}^{\alpha }=\sum_{\alpha ,k}I_{i,k}^{\alpha }. \end{array}$$

The interactions describe the exchanges of extensities and energy between the subsystems. A particular interaction is defined by specifying the extensity or energy fluxes at each contact point. This specification of an extensity flux and its accompanying energy flux allows later to easily construct the entire dynamics of the overall thermodynamic system. As each contact point *k* has a well-defined intensity $Y_{i,k}^{\alpha }$ for the extensity exchanged, the inflow of an extensity $J_{i,k}^{\alpha }$ into subsystem *i* is directly proportional to the carried energy flux

(6) $$\begin{array}{c} I_{i,k}^{\alpha }=Y_{i,k}^{\alpha }J_{i,k}^{\alpha } \end{array}$$

and thus, only one of the two needs to be specified. This connection can also be seen in the units of extensities and intensities: while the energy fluxes $I_{i,k}^{\alpha }$ always have the units of energy per time (power) $[I_{i,k}^{\alpha }]=W$, the intensity units $\left[Y_{i,k}^{\alpha }\right]=W/\left[J_{i,k}^{\alpha }\right]$.

The extensity fluxes between subsystems can be reversible or irreversible, and in specifying the respective fluxes at the contact points, the extent of the irreversibility is specified. Usually, the fluxes are given by transport equations, such as the often used Newtonian or Fourier heat conduction, but of course, such transport laws can also be quite complex, especially if multi-extensity fluxes occur.

Apart from entropy, all extensities of relevance in the Vuilleumier refrigerator are conserved in an interaction and thus, all corresponding extensity fluxes will balance at each moment. In an irreversible interaction, entropy is generated, and thus the net entropy outflow from an interaction is positive. Therefore, irreversible interactions require at least one of their contact points to be an entropy contact for disposing the produced entropy.

Finally “pure” energy fluxes are introduced for which the carrier extensity remains unspecified. These are used if one is only interested in the energy aspect of an interaction.

How those concepts work in detail will become apparent below in the modeling section for the Vuilleumier refrigerator.

### 2.2. The Endoreversible Vuilleumier Refrigerator Model

**Figure 2:**
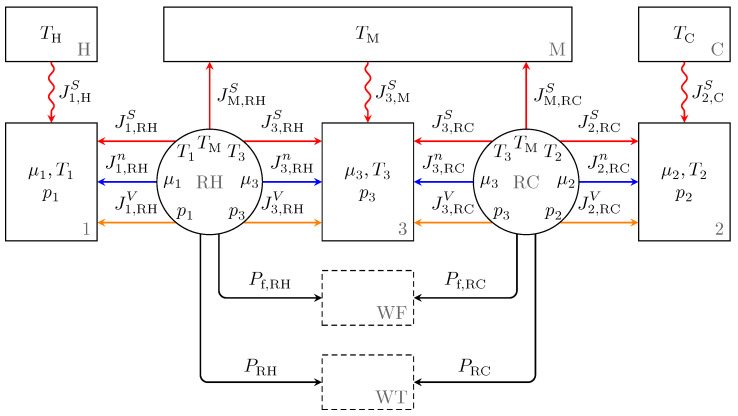
Endoreversible model of the Vuilleumier refrigerator (VMR). The rectangles with solid lines represent physical reservoirs, while the rectangles with dashed lines are bookkeeping reservoirs, which simplify the description. The circles represent engines with no reservoir functionality. Engines and reservoirs are connected by reversible (straight lines) and irreversible (wavy lines) interactions. In the top row, the three infinite capacity heat baths are located, where H delivers heat as an energy source for the VMR, C is the system to be cooled, and M is the environment into which the waste heat is dumped. The VMR also gets power (for instance electricity based) from WT, and WF collects the power loss due to frictional losses in the operation of the refrigerator. Reservoirs 1, 2, and 3 represent the working fluid in the three working spaces, and RH and RC are the engines describing the regenerators connected to the hot and cold VMR working spaces.

The three external heat baths H, M, and C, to which the Vuilleumier refrigerator is connected, are assumed to have constant temperatures $T_{H}$, $T_{M}$, and $T_{C}$, respectively. The endoreversible Vuilleumier refrigerator model we use is shown in Figure 2. It consists of three reservoirs, 1, 2, and 3, representing the working spaces. The two ideal regenerators are modeled as engines, and additionally, we have two bookkeeping reservoirs to account for the frictional losses (WF) and for the net power (WT), which must be provided from an auxiliary drive. All reservoirs and interactions are explained in more detail below.

### 2.3. The Working Fluid

In the three reservoirs representing the three cylinders, the thermodynamics is determined by the physical properties of the gas used as working fluid. Here, we have chosen to use an ideal gas with given molar heat capacity ${\hat{c}}_{V}R$, where ${\hat{c}}_{V}$ is the dimensionless specific heat capacity at constant volume (later chosen to be 5/2 corresponding to a dimer gas) and *R* is the universal gas constant. For the ideal gas, the thermal equation of state is

(7) $$\begin{array}{c} pV=nRT, \end{array}$$

where p,V,n and *T* are the pressure, volume, mole number, and temperature, respectively. The caloric equation of state is

(8) $$\begin{array}{c} U={\hat{c}}_{V}nRT, \end{array}$$

with *U* being the internal energy.

The internal energy can also be expressed in terms of its natural extensities: entropy *S*, volume *V* and mol number *n*. Before we do that, we introduce the entropy in its typical dependence on *V*, *T*, and *n* as

(9) $$S=nR\left({\hat{c}}_{V}\ln\frac{T}{T_{0}}+\ln\frac{V}{V_{0}}-\ln\frac{n}{n_{0}}\right)+n\frac{S_{0}}{n_{0}},$$

with reference temperature $T_{0}$, reference volume $V_{0}$, reference mole number $n_{0}$, and reference entropy $S_{0}\left(T_{0},V_{0},n_{0}\right)$. Then, from Equation (9), the temperature *T* is expressed as follows:

(10) $$T\left(S,V,n\right)={\left(\frac{V_{0}T_{0}^{{\hat{c}}_{V}}}{n_{0}}\frac{n}{V}\exp\left(\frac{S}{nR}-\frac{S_{0}}{n_{0}R}\right)\right)}^{\frac{1}{{\hat{c}}_{V}}}.$$

in terms of the extensities S,V and *n*. In combination with Equation (8), one finds the principle equation of state [58] to be:

(11) $$\begin{array}{c} U={\hat{c}}_{V}nR\;T\left(S,V,n\right)={\hat{c}}_{V}nR{\left(\frac{V_{0}T_{0}^{{\hat{c}}_{V}}}{n_{0}}\frac{n}{V}\exp\left(\frac{S}{nR}-\frac{S_{0}}{n_{0}R}\right)\right)}^{\frac{1}{{\hat{c}}_{V}}}. \end{array}$$

Using Equation (1), the functional dependencies of all intensities on the extensities *S*, *V*, and *n* can be obtained. In particular, one finds for the pressure of the fluid

(12) $$\begin{array}{c} p\left(S,V,n\right)=-{\left(\frac{\partial U}{\partial V}\right)}_{S,n}=\frac{nR}{V}T\left(S,V,n\right), \end{array}$$

and for the chemical potential of the fluid

(13) $$\begin{array}{c} \mu \left(S,V,n\right)={\left(\frac{\partial U}{\partial n}\right)}_{S,V}=\left({\hat{c}}_{V}R+R-\frac{S}{n}\right)T\left(S,V,n\right). \end{array}$$

### 2.4. Heat Transfer

For the Vuilleumier refrigerator as a heat-driven refrigerator, the heat transfer processes are crucial. These processes are a major source of irreversibilities and thus, lead to considerable entropy production. As depicted in Figure 2 by wavy lines, these heat transfers occur in the three heat fluxes between subsystems H and 1, and M and 3, as well as C and 2. Below, the following notation for extensity and energy fluxes is used: as there are only interactions coupling two contact points of two different subsystems, one can use the name of the subsystem from which a flux originates as the name for the contact point of the receiving subsystem. All heat transfers in the Vuilleumier refrigerator are modeled as Newtonian with a heat flux proportional to the temperature difference:

(14) $$\begin{array}{cc} I_{1,H}^{S} & =\kappa \left(T_{H}-T_{1}\right)=-I_{H,1}^{S}, \end{array}$$

(15) $$\begin{array}{cc} I_{3,M}^{S} & =\kappa \left(T_{M}-T_{3}\right)=-I_{M,3}^{S}, \end{array}$$

(16) $$\begin{array}{cc} I_{2,C}^{S} & =\kappa \left(T_{C}-T_{2}\right)=-I_{C,2}^{S}, \end{array}$$

where κ is the heat conductance (with units ${W\;K}^{-1}$), which here, for simplicity, is chosen to be equal for all three heat fluxes. The corresponding entropy fluxes are given by

(17) $$\begin{array}{cccc} J_{1,H}^{S} & =I_{1,H}^{S}/T_{1}, & \;\;\;J_{H,1}^{S} & =I_{H,1}^{S}/T_{H}, \end{array}$$

(18) $$\begin{array}{cccc} J_{3,M}^{S} & =I_{3,M}^{S}/T_{3}, & \;\;\;J_{M,3}^{S} & =I_{M,3}^{S}/T_{M}, \end{array}$$

(19) $$\begin{array}{cccc} J_{2,C}^{S} & =I_{2,C}^{S}/T_{2}, & \;\;\;J_{C,2}^{S} & =I_{C,2}^{S}/T_{C}. \end{array}$$

### 2.5. The Regenerators

The Vuilleumier refrigerator contains two regenerators, which strongly influence the efficiency of the refrigeration process. In general, if a fluid is to be heated and cooled cyclically, regenerators are a means to reduce entropy production and heat waste by storing and releasing heat at a range of temperatures. In order to put the focus on the heat conduction and frictional irreversibilities present in the operation of the Vuilleumier refrigerator, we have chosen to use ideal regenerators, in which such intermediate storage occurs without entropy production. For obtaining a simple model with very low numerical effort, ideal regenerators can be conveniently modeled as engines in the endoreversible framework; for more details, see [24]. The ideal regenerators themselves do not contain any working gas because the irreversibilties caused inside real regenerators are here intentionally excluded—thus, *ideal* regeneration.

The gas streams between reservoirs 1 and 3 passing through regenerator RH as well as the gas streams between 3 and 2 going through RC are multi-extensity fluxes [49], as they comprise a mol number flux as well as an entropy flux. The mol number transport is chosen to depend on the pressure difference between the respective reservoirs:

(20) $$\begin{array}{cc} J_{1,\mathrm{RH}}^{n} & =-J_{\mathrm{RH},1}^{n}=\alpha (p_{3}-p_{1})=-J_{3,\mathrm{RH}}^{n}=J_{\mathrm{RH},3}^{n}, \end{array}$$

(21) $$\begin{array}{cc} J_{2,\mathrm{RC}}^{n} & =-J_{\mathrm{RC},2}^{n}=\alpha (p_{3}-p_{2})=-J_{3,\mathrm{RC}}^{n}=J_{\mathrm{RC},3}^{n}. \end{array}$$

With these flux definitions, it is apparent that the mol number inside the regenerator remains zero and thus fulfills Equation (4) for the extensity mol number. Here, α is the mass transport coefficient.

From the mol number flux entering reservoir *i*, we can immediately derive the entropy flux by using the molar entropy $S_{m,i}=S_{i}/n_{i}$ of reservoir *i* here shown for reservoirs 1 and 2:

(22) $$\begin{array}{cc} J_{1,\mathrm{RH}}^{S} & =S_{m,1}J_{1,\mathrm{RH}}^{n}, \end{array}$$

(23) $$\begin{array}{cc} J_{2,\mathrm{RC}}^{S} & =S_{m,2}J_{2,\mathrm{RC}}^{n}. \end{array}$$

The other entropy fluxes follow the same scheme.

Just as for the mol number, one has to insure also for entropy that Equation (4) is fulfilled for each of the regenerators. In order to achieve this, there is an additional reversible entropy flux between each regenerator and the medium heat bath. We suppose that this bath allows to receive or discard entropy at no cost, as it is representing the environment. The resulting entropy fluxes $J_{\mathrm{RH},M}^{S}$ and $J_{\mathrm{RC},M}^{S}$ are determined from

(24) $$\begin{array}{cc} 0 & =J_{\mathrm{RH},M}^{S}+J_{\mathrm{RH},1}^{S}+J_{\mathrm{RH},3}^{S}, \end{array}$$

(25) $$\begin{array}{cc} 0 & =J_{\mathrm{RC},M}^{S}+J_{\mathrm{RC},2}^{S}+J_{\mathrm{RC},3}^{S}, \end{array}$$

As already pointed out above, another important loss term considered in the Vuilleumier refrigerator model stems from the frictional losses when the two pistons with their regenerators are moved during the cyclic operation. These losses are represented by (pure) energy fluxes from each regenerator to the bookkeeping reservoir WF. From there, this energy can be dissipated to heat and delivered, for instance, to the environment. For our analysis, the important point is that one can quantify these losses. Here, we assume that the lost energy is proportional to the velocity of the piston squared. With a constant cylinder diameter, this can be expressed in terms of the volume changes and is here chosen to be

(26) $$\begin{array}{c} P_{f,i}=\beta {\dot{V}}_{i}^{2}, \end{array}$$

where β is the mechanical friction coefficient [59].

Finally, one has to ensure that the energy balance is kept at zero for each instant of time. This is achieved by collecting the net energy flows $P_{1}$ and $P_{2}$ from the two regenerators in the bookkeeping reservoir WT. The net energy flows are determined from Equation (5) resulting in

(27) $$\begin{array}{cc} P_{\mathrm{RH}} & =-P_{f,\mathrm{RH}}+I_{\mathrm{RH},M}^{S}+I_{\mathrm{RH},1}^{S}+I_{\mathrm{RH},3}^{S}+I_{\mathrm{RH},1}^{n}+I_{\mathrm{RH},3}^{n}+I_{\mathrm{RH},1}^{V}+I_{\mathrm{RH},3}^{V}, \end{array}$$

(28) $$\begin{array}{cc} P_{\mathrm{RC}} & =-P_{f,\mathrm{RC}}+I_{\mathrm{RC},M}^{S}+I_{\mathrm{RC},2}^{S}+I_{\mathrm{RC},3}^{S}+I_{\mathrm{RC},2}^{n}+I_{\mathrm{RC},3}^{n}+I_{\mathrm{RC},2}^{V}+I_{\mathrm{RC},3}^{V}. \end{array}$$

Notice that the volume fluxes $J_{\mathrm{RH},1}^{V}$, etc., are not yet defined. However, once they are known by specifying the time dependence of the volumes, the complete dynamics of the Vuilleumier refrigerator and thus, the resulting energy flows can be determined.

In case a more detailed description of the regenerators is required that still leads to relatively low numerical effort and few degrees of freedom, the endoreversible regenerator models developed and validated in [27,60] can be applied.

### 2.6. The Dynamics

The dynamics of the Vuilleumier refrigerator is set by the external controls for the volume changes and a coupled set of differential equations for the temporal development of the thermodynamic variables in the reservoirs. The latter are obtained from Equation (2) and Equation (3) for reservoirs 1 and 2:

(29) $$\begin{array}{cc} {\dot{S}}_{1} & =J_{1,H}^{S}+J_{1,\mathrm{RH}}^{S}=\kappa \left(T_{H}-T_{1}\right)/T_{1}+S_{m,1}{\dot{n}}_{1}, \end{array}$$

(30) $$\begin{array}{cc} {\dot{V}}_{1} & =J_{1,\mathrm{RH}}^{V}, \end{array}$$

(31) $$\begin{array}{cc} {\dot{n}}_{1} & =J_{1,\mathrm{RH}}^{n}=\alpha (p_{3}-p_{1}), \end{array}$$

(32) $$\begin{array}{cc} {\dot{S}}_{2} & =J_{2,C}^{S}+J_{2,\mathrm{RC}}^{S}=\kappa \left(T_{C}-T_{2}\right)/T_{2}+S_{m,2}{\dot{n}}_{2}, \end{array}$$

(33) $$\begin{array}{cc} {\dot{V}}_{2} & =J_{2,\mathrm{RC}}^{V}, \end{array}$$

(34) $$\begin{array}{cc} {\dot{n}}_{2} & =J_{2,\mathrm{RC}}^{n}=\alpha (p_{3}-p_{2}), \end{array}$$

as well as for reservoir 3:

(35) $$\begin{array}{cc} {\dot{S}}_{3} & =J_{3,M}^{S}+J_{3,\mathrm{RC}}^{S}+J_{3,\mathrm{RC}}^{S}=\kappa \left(T_{M}-T_{3}\right)/T_{3}-S_{m,3}{\dot{n}}_{1}-S_{m,3}{\dot{n}}_{2}, \end{array}$$

(36) $$\begin{array}{cc} {\dot{V}}_{3} & =J_{3,\mathrm{RH}}^{V}+J_{3,\mathrm{RC}}^{V}=-(J_{1,\mathrm{RH}}^{V}+J_{2,\mathrm{RC}}^{V}), \end{array}$$

(37) $$\begin{array}{cc} {\dot{n}}_{3} & =J_{3,\mathrm{RH}}^{n}+J_{3,\mathrm{RC}}^{n}=\alpha \left(p_{1}-p_{3}\right)+\alpha \left(p_{2}-p_{3}\right). \end{array}$$

## 3. Refrigerator Controls: The AS Motion Class

The aim of this research activity is to determine potential performance improvements for a Vuilleumier refrigerator by modifications of its piston motion. While in principle this goal can be achieved by performing a control theory based analysis, as done in [26], this route is connected with extensive numerical effort. For our desired estimation of the performance potential, another less numerically expensive approach is available that is based on the AS (“adjustable sinusoidal”) motion class for cyclic dynamics [24].

The AS motion class consists of two parameter periodic functions, which can capture two important features of a periodic movement: the fraction of time spent above and below 1/2, which is controlled by δ, and the fraction of time spent close to its extreme values, which is controlled by σ. It is given by

(38) $$\begin{array}{c} f_{\mathrm{AS}}\left(x;\sigma ,\delta \right)=f_{1}\left(f_{2}\left(x;\delta \right);\sigma \right), \end{array}$$

where the two functions $f_{1}$ and $f_{2}$ are defined as

(39) $$\begin{array}{c} f_{1}\left(x;\sigma \right)=\left(\sin(2\pi x+\sigma \sin(4\pi x))+1\right)/2 \end{array}$$

and

(40) $$\begin{array}{c} f_{2}\left(x;\delta \right)=x+\delta \left(1-\cos\left(2\pi x\right)\right), \end{array}$$

respectively. Notice that the standard sin function scaled to values between 0 and 1 is recovered for σ=δ=0.

**Figure 3:**
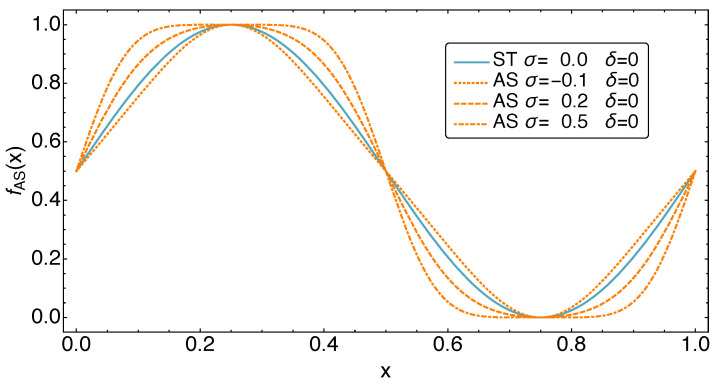
The shape of the AS motion class for different values of the parameter σ. One finds the common sinusoidal behavior at σ=0. Values σ<0 modify the motion towards a more triangular shape, i.e., decrease the time fraction spent at extremal positions. In contrast, values σ>0 cause a more square-like shape, i.e., increase the time fraction spent at extremal positions.

**Figure 4:**
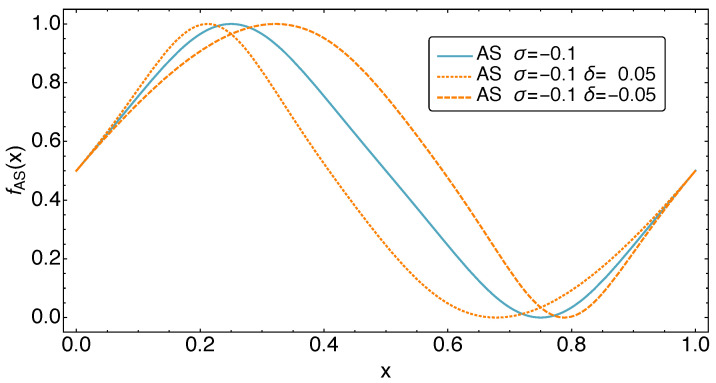
The shape of the AS motion class for different values of the parameter δ, which controls the time fraction spent above and below 1/2. One finds equal times spent within both regions at δ=0.

The effects of the dimensionless parameters σ and δ on the functional form of the periodic motion is demonstrated in Figure 3 and Figure 4. Note that the parameters σ and δ are limited to certain parameter ranges in order to lead to the desired form changes of $f_{\mathrm{AS}}$. These are −0.13<σ<0.6 and −0.08<δ<0.08.

Based on the AS motion class, the volume dynamics of the three working spaces is set to be

(41) $$\begin{array}{cc} V_{1}\left(t\right) & =V_{0}+Df_{\mathrm{AS}}\left(t/t_{0};\sigma_{1},\delta_{1}\right), \end{array}$$

(42) $$\begin{array}{cc} V_{2}\left(t\right) & =V_{0}+Df_{\mathrm{AS}}\left(t/t_{0}+\Delta ;\sigma_{2},\delta_{2}\right), \end{array}$$

(43) $$\begin{array}{cc} V_{3}\left(t\right) & =V_{0}+D\left(1-f_{\mathrm{AS}}\left(t/t_{0};\sigma_{1},\delta_{1}\right)\right)+D(1-f_{\mathrm{AS}}\left(t/t_{0}+\Delta ;\sigma_{2},\delta_{2}\right), \end{array}$$

where $t_{0}$ is the cycle time, $V_{0}$ the dead volume, *D* the displacement, and Δ an additional parameter, which results in a time shift between the sinusoidal motion of the two displacer pistons. Thus, the complete AS motion is described by five dimensionless parameters: $\sigma_{1}$, $\delta_{1}$, $\sigma_{2}$, $\delta_{2}$, and Δ. For the standard harmonic dynamics of the Vuilleumier refrigerator, one uses a phase shift of π/2, which corresponds to Δ=0.25 and $\sigma_{1}=\delta_{1}=\sigma_{2}=\delta_{2}=0$. From this volume dynamics, the corresponding volume fluxes can be easily determined as

(44) $$\begin{array}{c} J_{1,\mathrm{RH}}^{V}={\dot{V}}_{1}\left(t\right)=-J_{\mathrm{RH},1}^{V}, \end{array}$$

(45) $$\begin{array}{c} J_{2,\mathrm{RC}}^{V}={\dot{V}}_{2}\left(t\right)=-J_{\mathrm{RC},2}^{V}, \end{array}$$

and so forth. Note that $V_{3}$ follows from the overall conservation of volume.

## 4. Performance Measures

In order to study the performance changes of the Vuilleumier refrigerator with varying volume controls, two performance measures are employed: the cooling power and a COP suitably defined. First, the cycle averaged cooling power is introduced as follows:

(46) $$\begin{array}{c} q_{C}=\frac{1}{t_{0}}\int_{0}^{t_{0}}I_{2,C}^{S}\;dt. \end{array}$$

The cooling power $q_{C}$ is generated through an energy input from two sources: the main source is the heat input from heat bath H, and additionally, work input from the auxiliary drive may contribute. The cycle averaged heat flux from H is given by

(47) $$\begin{array}{c} q_{H}=\frac{1}{t_{0}}\int_{0}^{t_{0}}I_{1,H}^{S}\;dt, \end{array}$$

and the cycle averaged auxiliary power input by

(48) $$\begin{array}{c} P_{\mathrm{aux}}=-\frac{1}{t_{0}}\int_{0}^{t_{0}}P_{\mathrm{RH}}+P_{\mathrm{RC}}\;dt. \end{array}$$

Based on these cycle averaged energy fluxes, we define an exergetic COP, in which the heat input and the work input are measured on equal exergetic footing by using the Carnot efficiency $\eta_{C}=1-T_{M}/T_{H}$ for a heat engine operating between the hot heat bath H and the intermediate bath M, as follows:

(49) $$\begin{array}{c} \mathrm{COP}=\frac{q_{C}}{\eta_{C}q_{H}+P_{\mathrm{aux}}}. \end{array}$$

## 5. Results

The goal of this research effort is to obtain good estimates for the potential performance increase of a Vuilleumier refrigerator by optimally controlling the volume changes in its three working spaces. The increase is measured with respect to the standard harmonic motion, which below is labeled with “ST”, while the optimal motion within the AS class is labeled with “OS” (optimized sinusoidal). The optimal motion within the AS class is found by numerically varying the motion parameters $\sigma_{1},\delta_{1},\sigma_{2},\delta_{2}$ and Δ until an optimum is achieved. This task is performed by a variant of the Nelder–Mead algorithm [61].

Below, the performance results for maximizing the cooling power of a Vuilleumier refrigerator are presented. For that, we chose refrigerator parameters which describe a 10 kW refrigerator. In particular, the parameters are chosen as follows: $T_{H}=300{\;}^{\circ }C$, $T_{M}=25{\;}^{\circ }C$, $T_{C}=0{\;}^{\circ }C$, $t_{0}=0.1\;s$, $n_{0}=5\;\mathrm{mol}$, $V_{\mathrm{dead}}=0.1\;L$, ΔV=1 L, ${\hat{c}}_{V}=5/2$. Our endoreversible Vuilleumier refrigerator model features three additional parameters, which characterize the transport properties for the heat conduction, the gas transport through the regenerators, and the frictional losses when moving the pistons: $\kappa_{0}=2\times 10^{4}\;{W\;K}^{-1}$, $\alpha_{0}=300\;{\mathrm{mol s}}^{-1}{\mathrm{bar}}^{-1}$, and $\beta_{0}=10\;kJs/m^{6}$. These are chosen such that with their above “base” values a “base case” is set for which the cooling power does not increase much further if κ and α are increased. This base case will serve as a comparison, when in a later part of our investigation these parameters are varied to determine their influence on the refrigerator performance.

### 5.1. Optimized Piston Motion

In this section, the optimized piston motion for maximizing the cooling power of the Vuilleumier refrigerator is presented with $\kappa_{0}=5\times 10^{4}\;{W\;K}^{-1}$, $\alpha_{0}=100\;{\mathrm{mol s}}^{-1}{\mathrm{bar}}^{-1}$, and $\beta_{0}=10kJs/m^{6}$. These values are close to the base case values, but allow better readable dynamics figures.

**Figure 5:**
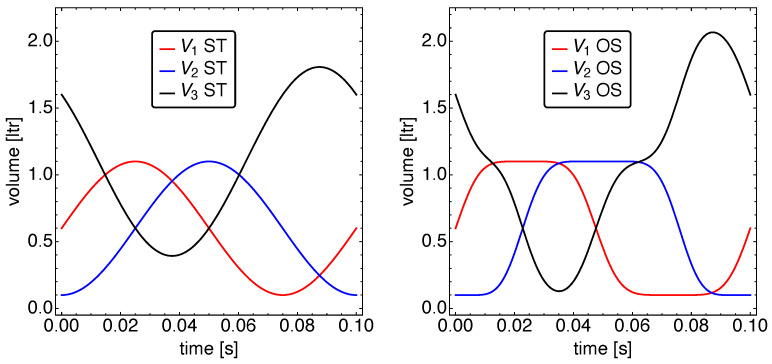
The volume dynamics V(t) for standard motion (**left**) and optimized motion (**right**). Notice that for the optimized motion, both pistons spent more time at positions close to their extrema.

In Figure 5, the volume dynamics is shown in comparison to the standard motion. It is immediately apparent that the cold and, even more so, the hot working space make use of the possibility to spend more time close to their extreme volumes. In particular, the hot volume spends more than 0.2 s very close to $V_{1}=0.1\;L$ and $V_{1}=1.1\;L$ each. For $V_{3}$, this is different, as the combination of the changes in $V_{1}$ and $V_{2}$ leads to relatively straight constant velocity stretches. Moreover, the minimum and maximum values for $V_{3}$ are much closer to the theoretically possible extreme values $V_{3}=0.1\;L$ and $V_{3}=2.1\;L$. A sizable difference in the times spent below and above the respective mean volumes cannot be observed.

It is interesting to note that this optimized volume dynamics is very similar to the “D-motion” considered in [28,29] for a free-piston Vuilleumier heat pump. Moreover, the respective displacer piston motion is also rather similar to the optimal working piston motion of the alpha-Stirling engines investigated in [24,26].

**Figure 6:**
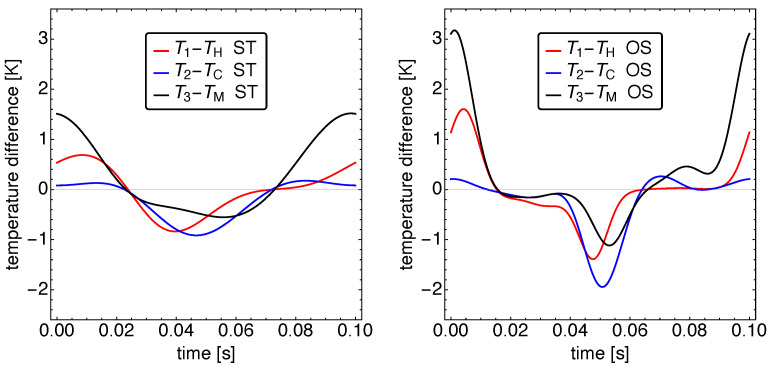
The resulting dynamics of the temperature differences between the working fluid in the working spaces and the corresponding heat baths for standard motion (**left**) and optimized motion (**right**). Notice the larger temperature differences between the heat sources/sinks and the working gas in the respective cylinders for optimized motion. This causes larger heat transfer and thus, increased cooling power.

The resulting temperature dynamics is shown in Figure 6. Here, the temperature differences between the working fluid in the working spaces and the corresponding heat baths are presented, which allows an immediate recognition of the heat flow direction. An apparent yet surprising fact is that practically all three heat flows change their direction at the same times. Thus, the Vuilleumier refrigerator has essentially two major states of either taking in heat from all baths or losing heat to all baths. While for the standard motion one can still recognize a roughly sinusoidal behavior, this is clearly different for the OS motion. For the OS motion, there are four phases: two with pronounced larger temperature differences than for the ST motion, and two phases with relatively small differences. An interesting observation is that the cold working space temperature never becomes much larger than the cold bath temperature, and thus, there is never a sizable back flow of heat from the refrigerator into the cold bath.

**Figure 7:**
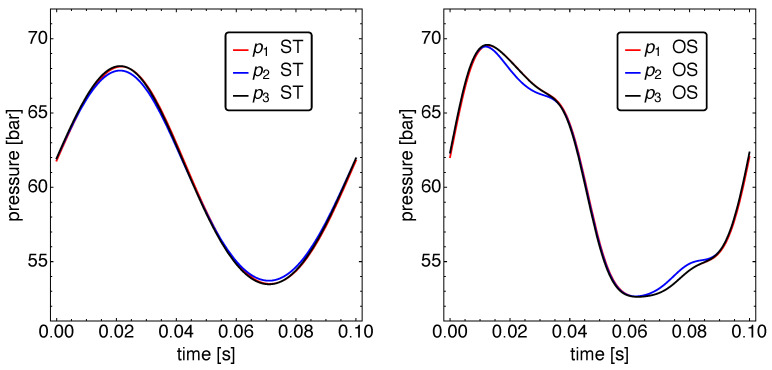
The pressure dynamics p(t) for standard motion (**left**) and optimized motion (**right**). Notice that the pressure differences between the three cylinders almost vanish because the gas flow resistance of the regenerators is low.

Finally, in Figure 7, the pressure dynamics is shown. For the ST as well as for the OS motion the pressure in all three refrigerator working spaces are relatively close together, which is a direct consequence of the α value used for the working fluid transport through the regenerators.

Only the cold working space pressure differs a bit more from the other two compartments for both the ST and OS motion. There is, however, a clear difference in the form of the pressure graphs. While the ST dynamics features a near harmonic behavior, the OS pressures are oscillating between more extreme values, and the corresponding graphs resemble more a square wave with inclined top and bottom parts. For the OS motion, the pressure extrema are about 2 bar higher and 1 bar smaller than for the ST case. A surprising feature is that the pressure extrema correlate strongly with the vanishing heat fluxes. This is because the Vuilleumier machine operates based on thermal compression and expansion. During the phase where the displacer movements cause a drop in the overall mean gas temperature, the pressure drops. This leads to reductions in the individual working space temperatures and in turn, induces heat fluxes from the heat baths. As opposed to that, during the phase where the displacer movements cause a rise in the overall mean gas temperature, the pressure increases. This leads to rising individual working space temperatures and induces heat fluxes out of the working spaces into the heat baths.

**Figure 8:**
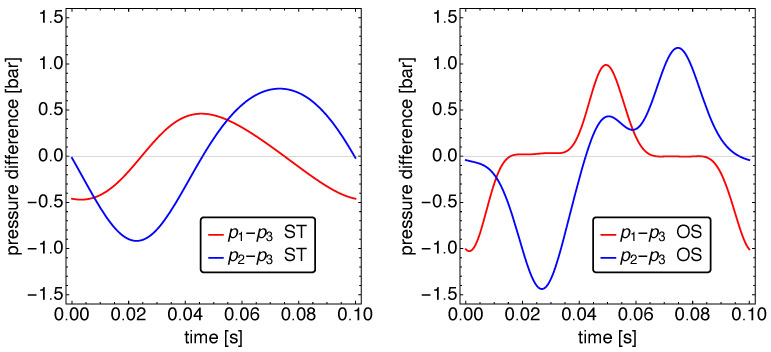
The pressure dynamics Δp(t) between adjacent cylinders closely resembles the motion speed of the respective regenerators.

In order to obtain a better view on the pressure dynamics, we plot in Figure 8 the pressure difference of the hot and cold working spaces with respect to the medium working space. The graphs show clearly the larger pressure difference of the cold working space. While for the ST motion, the pressure differences show a close to harmonic behavior, this is strongly different for the OS motion. The hot pressure difference shows—similar to the temperature difference—a four-phase behavior: two pronounced peaks and two close to zero in-between phases. All are of roughly the same duration. The cold pressure difference looks similar; however, in the phase between the negative and positive pressure difference, a small intermediate maximum can be seen. During that maximum, working space 3 receives gas from both of the other working spaces, while during the other time, only one of those working spaces has a noticeable gas exchange with working space 3. This is clearly different for the ST motion.

### 5.2. Optimized Piston Motion: Friction

In this section, the dependence of the cooling power on the size of the frictional losses is studied. We thus look at the performance measures cooling power and exergetic COP as a function of the friction coefficient β. Before we do that, we look at the two energy sources and their contributions to the operation of the Vuilleumier refrigerator in order to restrict our analysis to those values of β, for which the power input is small enough compared to the heat input so that one can still speak of an auxiliary external drive. Here, that restricts β to the range of $\beta <400\;kJs/m^{6}$.

**Figure 9:**
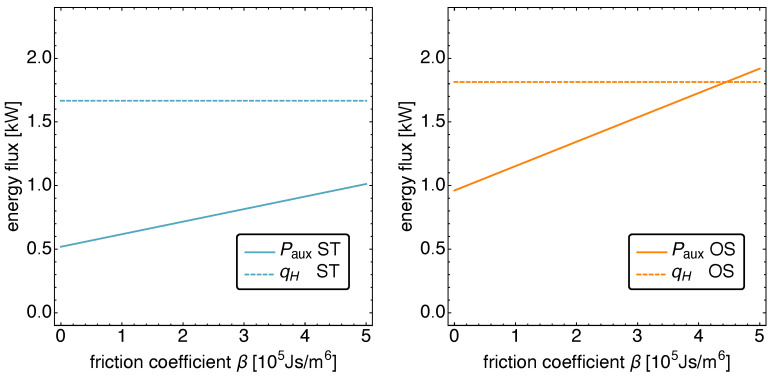
The power input $P_{\mathrm{aux}}$ and the heat input $q_{H}$ versus the friction coefficient β for standard motion (**left**) and optimized motion (**right**). Notice the linear increase in the necessary power input in both cases while the heat input remains constant.

In Figure 9, the power input $P_{\mathrm{aux}}$ and the heat input $q_{H}$ are plotted as a function of β for the ST and the OS motion. It is apparent that the heat input is independent of β, while the auxiliary power $P_{\mathrm{aux}}$ shows a linear increase with the friction coefficient. This is a direct consequence of the performance goal “cooling power”: the optimal motion as well as the standard motion are the same, independent of the size of β. Thus, the thermodynamic fluxes remain the same, and only the power input is increased to compensate for the increasing friction.

**Figure 10:**
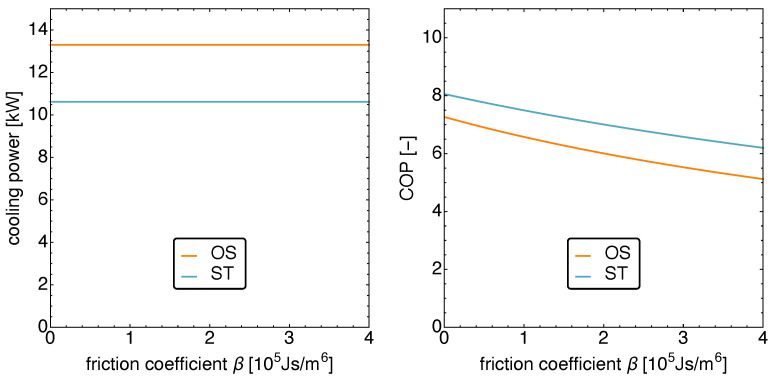
The cooling power $q_{C}$ (**left**) and the COP (**right**) versus the friction coefficient β for standard motion and optimized motion. Notice that the cooling power remains constant, while the COP decreases with increasing friction. Further note the lower COP for optimized motion, compared to standard motion, which is a direct consequence of the optimization objective.

It is therefore not surprising that the cooling power stays constant independent of the friction as shown on the left in Figure 10. The interesting feature one learns from that figure is that the OS motion leads to an increase in cooling power of about 26%.

The right panel in Figure 10 depicting the exergetic COP shows two features of relevance. Not surprisingly, with increasing friction the COP decays. What might come as a surprise is that the COP of the ST motion is larger than that for the OS motion. However, considering that the optimization objective is the cooling power and not the COP, this becomes understandable.

### 5.3. Optimized Piston Motion: Heat Conduction

We now turn to the influence of the heat conduction coefficient κ on the performance of the Vuilleumier refrigerator. Again, the range of κ values for which the main energy source is heat from the hot heat bath is determined first.

**Figure 11:**
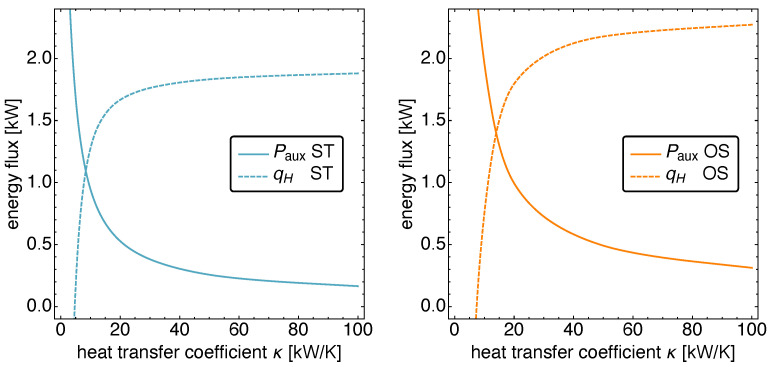
The power input $P_{\mathrm{aux}}$ and the heat input $q_{H}$ versus the heat conduction coefficient κ for standard motion (**left**) and optimized motion (**right**). Notice the decrease in power input and the simultaneous increase in heat input with increasing heat conduction coefficient. For values $\kappa <20\;{\mathrm{kW K}}^{-1}$, the power input is no longer auxiliary but represents the main input power source.

Figure 11 shows the power input $P_{\mathrm{aux}}$ and the heat input $q_{H}$ for ST and OS motion. It is apparent that for $\kappa <20\;{\mathrm{kW K}}^{-1}$, the auxiliary character of the power input is lost, and thus, we restrict ourselves to the range $20\;{\mathrm{kW K}}^{-1}<\kappa <100\;{\mathrm{kW K}}^{-1}$.

**Figure 12:**
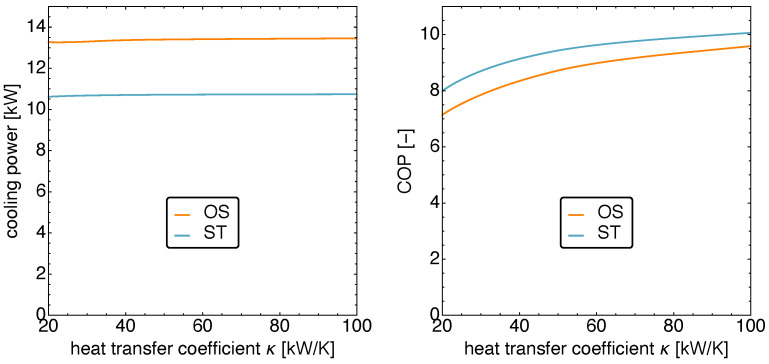
The cooling power $q_{C}$ (**left**) and the COP (**right**) versus the heat conduction coefficient κ for standard motion and optimized motion. Notice that the cooling power remains mostly constant, while the COP increases with the heat conduction coefficient.

In that range, one finds that the cooling power does not depend significantly on κ as can be seen on the left of Figure 12.

On the right in Figure 12, the corresponding COPs are shown. As in the previous study of the β dependence, here again the COP is higher for the ST than for the OS motion. The difference is not large and in the 5% range. However, both show an increase of about 25% with an increase in the coupling to the heat bath from $\kappa =20\;{\mathrm{kW K}}^{-1}$ to $\kappa =100\;{\mathrm{kW K}}^{-1}$.

### 5.4. Optimized Piston Motion: Mass Transport

Finally, the impact of the mass transport coefficient α on the Vuilleumier refrigerator performance is investigated. The mass transfer coefficient determines the regenerator’s resistance against the gas flow passing through it, and thus, influences what pressure differences occur between the working spaces.

**Figure 13:**
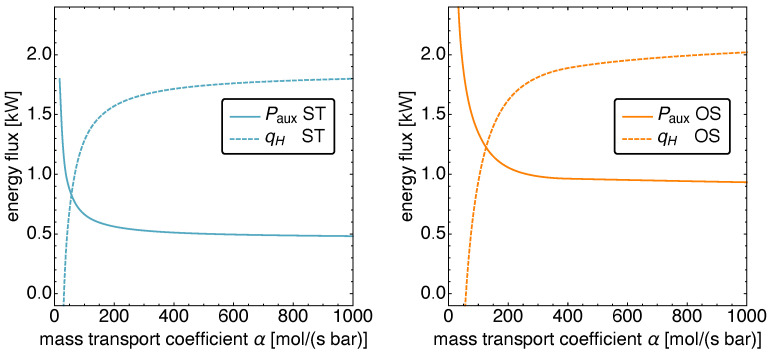
The power input $P_{\mathrm{aux}}$ and the heat input $q_{H}$ versus the mass transport coefficient α for standard motion (**left**) and optimized motion (**right**). Notice the decrease in power input and the simultaneous increase in heat input with the increasing mass transport coefficient. For values $\alpha <100\;{\mathrm{mol s}}^{-1}{\mathrm{bar}}^{-1}$, the power input is no longer auxiliary but represents the main input power source.

Figure 13 displays the auxiliary power input $P_{\mathrm{aux}}$ and the heat input $q_{H}$ as functions α. In the range $\alpha <200\;{\mathrm{mol s}}^{-1}{\mathrm{bar}}^{-1}$, one finds a strong dependence, while for larger α values, the dependence becomes weaker and weaker. We thus restrict ourselves to values $100\;{\mathrm{mol s}}^{-1}{\mathrm{bar}}^{-1}<\alpha <1000\;{\mathrm{mol s}}^{-1}{\mathrm{bar}}^{-1}$.

**Figure 14:**
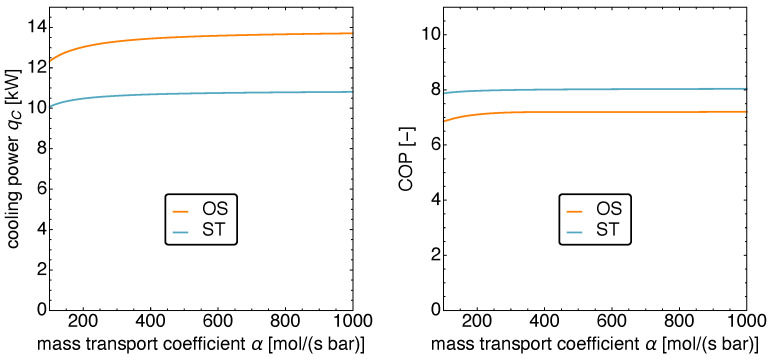
The cooling power $q_{C}$ (**left**) and the COP (**right**) versus the mass transport coefficient α for standard motion and optimized motion. Notice that the cooling power decreases for values $\alpha <200\;{\mathrm{mol s}}^{-1}{\mathrm{bar}}^{-1}$, while it remains mostly constant otherwise. The COP increases with the mass transport coefficient.

On the left of Figure 14, the resulting cooling power is shown. For large α, it stays in the known range of 14kW for the OS motion. For smaller values however, the cooling power starts to decay and when α is at its minimum value allowed here, the cooling power is reduced by about 10%. The cooling power for the ST motion behaves similarly, even though its decay is smaller.

Finally, on the right in Figure 14 the corresponding COPs are shown. Both are very close together and do not change over the range of investigated α values at all.

## 6. Conclusions

We modeled a Vuilleumier refrigerator by means of Endoreversible Thermodynamics and optimized the motion of its regenerator pistons based on the adjustable sinusoidal (AS) motion class. The optimization results showed that the cooling power can be increased by up to 15% by applying an optimized motion of the regenerators, compared to the standard motion. Additionally, the reduction in mechanical friction is always advisable to reduce the power required for the mechanical drive. Interestingly, the mechanical friction does, however, not interfere with the thermal process itself. Furthermore, taking measures to increase the heat transfer coefficient is advisable to reduce the power required for the mechanical drive for the cost of larger (waste) heat consumption, while the cooling power remains almost constant. Finally, one should seek to increase the mass transfer coefficient—i.e., reduce the mass flow resistance of the regenerator—for the same reason as above. In this analysis using the cooling power as the optimization objective is particularly justified, because the intended application is the recovery of waste heat, which can be considered to be a basically “cost-free” resource. Thus, the most cost-effective application of such a device implies the generation of the maximum possible cooling power for a certain amount of resources used to build the device itself.
